# Pushing forward: Understanding physical activity in adults with medical complexity

**DOI:** 10.1016/j.hctj.2025.100102

**Published:** 2025-04-18

**Authors:** Valerie Perkoski, Mary Shotwell, Charlotte Chatto, Judy Chandler

**Affiliations:** a Rocky Mountain University of Health Professions, 1800 South Novell Place, Provo, UT 84606, USA; b University of the Cumberlands, 6178 College Station Dr, Williamsburg, KY 40769, USA; c Central Michigan University, 1200 S. Franklin St, Mount Pleasant, MI 48859, USA

**Keywords:** Intellectual disability, Transitions, Physical activity, Ecological model

## Abstract

**Background:**

Adults with severe and profound intellectual disabilities (SPIDs) often encounter more significant healthcare needs than those without disabilities. People with SPIDs are more likely to have mobility impairments (MIs), yet little is known about physical activity (PA) experiences among those with SPIDs and MIs once they transition out of pediatric and school-based settings. This study explores the experience of PA in adults with SPIDs and MIs based on clinician perspectives.

**Methods:**

Eight clinicians engaged in a semi-structured interview and described their experiences with PA in adults with SPIDs/MIs. Interviews were analyzed to determine common themes, and a reflexivity journal and field notes were used to corroborate and supplement findings. Data was organized according to the 5 socio-ecological model (SEM) levels and 16 a priori themes (1) intrapersonal: attitudes, physical factors, knowledge, and values, (2) interpersonal: supports outside the home, supports within the home, and home environment considerations, (3) organizational: disability-inclusive organizations, academic institutions, and medical institutions, (4) community: environment and priorities, and (5) policy: home and community-based services (HCBS), financial, academic and programming, and accessibility policies. Clinician-identified barriers and facilitators to PA were grouped under these 16 themes.

**Results:**

The 5 most prevalent PA facilitators included (1) PA preferences as uniquely individualized, (2) organizations providing PA for adults with multiple disabilities, (3) building, outdoor, and transportation accessibility, (4) the importance of allyship and socialization among those with SPIDs/MIs and between caregivers, and (5) advocacy for promoting monies toward prevention instead of illness. The 5 most prevalent barriers to PA were (1) building, outdoor, and transportation inaccessibility, (2) necessity of education on needs and opportunities for PA, (3) diagnosis, bodily structure, weakness, or pain in adults with SPIDs/MIs, (4) lack of competitive billing structure to support PA programs or clinician reimbursement, and (5) lack of HCBS education and support.

**Implications:**

Recognizing the interplay of each SEM level and factors influencing PA engagement may improve access and health outcomes among adults with SPIDs/MIs. Clinicians play a significant role in assessing, educating, and promoting PA opportunities for people with disabilities as they transition into and age within adult and community settings.

## Highlights

• Adults with severe and profound intellectual disabilities and mobility impairments face significant healthcare needs.
• This study explores their physical activity (PA) experiences post-transition from pediatric and school-based settings.
• Eight clinicians were interviewed. Their insights were analyzed using a socio-ecological model across five levels.
• Facilitators included PA preferences, supportive organizations, accessible environments, socialization, and advocacy.
• Barriers included inaccessibility, lack of PA education, physical limits, and billing issues.
• Insufficient Home and Community-Based Services (HCBS) education was also a significant barrier.
• Clinicians play a critical role in promoting and facilitating PA in adult settings.

## Graphical Abstract

**Figure:**
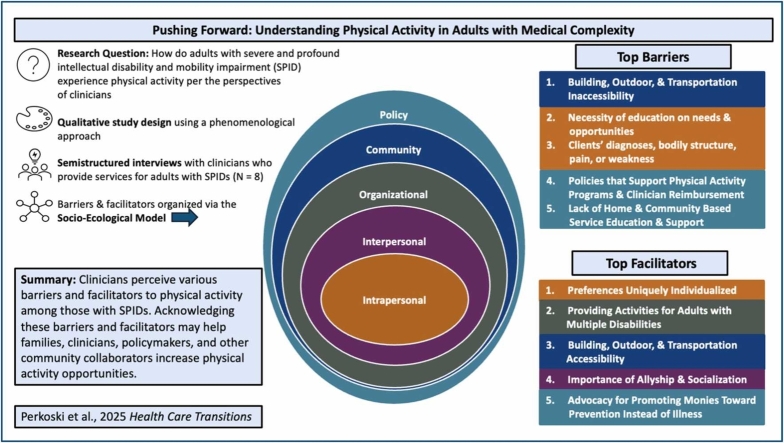
Barriers and Facilitators to Physical Activity Among Adults with Severe and Profound Intellectual Disabilities and Mobility Impairments from the Perspectives of Clinicians

## 1 Introduction

The U.S. Department of Education^1^ defines an intellectual disability (ID) as having intellectual functioning significantly below average during the developmental period. IDs are associated with deficits in adaptive behavior and may impact the individual’s educational performance.^1^ ID includes 4 levels defined by an individual's adaptive functioning in 3 domains (social, conceptual, and practical). These 4 ID levels include mild, moderate, severe, and profound.^2^ Developmental disabilities (DDs) are chronic disabilities that may be intellectual, physical, or both. These conditions are diagnosed before age 22 and may include visual or hearing impairments and cerebral palsy. An intellectual and developmental disability (IDD) is a simplified term that characterizes individuals with an ID with other DDs.^3^

Individuals with severe and profound intellectual disabilities (SPIDs) are more prone to physical conditions such as epilepsy, visual impairments, and muscular spasticity.^4^ Individuals with severe ID and mobility impairment (MI) have significantly lower bone quality, mainly due to reduced physical abilities and a lack of physical activity (PA).^5^ No data exists on the prevalence of those with SPIDs who are non-ambulatory; however, those with SPIDs are more likely to have MIs than those with mild and moderate intellectual disabilities (MMIDs).^6^

According to the U.S. Department of Health and Human Services (U.S. DHHS)^7^, adults should get 150–300 minutes per week of moderate-intensity PA or 75–150 minutes per week of vigorous-intensity aerobic activity. The World Health Organization (WHO)^8^ defines moderate-intensity activity as requiring an observable heart rate acceleration and a vigorous-intensity activity that causes rapid breathing and a substantial increase in heart rate. The U.S. DHHS also recommends that adults engage in muscle-building activities at least two days per week.^7^ For individuals with disabilities who cannot meet PA recommendations, the U.S. DHHS states that it is essential to participate in PA per their abilities as much as possible and to eliminate inactivity.^7^ It is important to note that no known PA guidelines are specific for people with SPIDs or SPIDs with MIs.

Individuals with SPIDs require PA programming in school-based settings. The Individuals with Disabilities Education Act (IDEA) mandates that children and young adults between the ages of 3–21 who have specific disabilities or developmental delays receive opportunities for physical education as part of an Individualized Education Program/Plan (IEP). In many U.S. states, once an individual with an IEP reaches the age of 22, they are no longer eligible for an IEP under IDEA.^9^ There is research on PA among children with disabilities^10^, ^11^, ^12^; however, there is limited evidence regarding PA in adults with IDs, specifically those with SPIDs/MIs once they transition out of school-based settings.

Various disciplines may provide care in the home or community, including occupational, physical, and speech therapists, behavioral health counselors, aids, and other clinicians, as well as staff members in adult day programs.^13^ As part of their intake or initiation of care, clinicians serving people with SPIDs in home or community-based environments may observe or perform assessments to evaluate the individual’s physiology and physical abilities that might influence engagement in PAs. For example, adapted PA experts offer encouragement and support to promote PA in adults with SPIDs. Examples of adapted PA experts include certified adapted physical education teachers, recreation program developers, therapeutic recreation specialists, certified inclusive fitness trainers, and coaches for athletes with disabilities.^14^ Adapted PA experts work in a variety of community settings and encourage PA via a variety of different modalities. For example, therapeutic recreation specialists may work in home-based environments or adult day facilities and assist with PA promotion via adapted sports, aquatics, and dance/movement interventions.^15^

Both occupational therapy (OT) and physical therapy (PT) practice include evaluations and interventions that may impact overall PA functioning as related to movement-focused activities. PTs may assist those with SPIDs/MIs in the community and home-based environments. Physical therapists create and monitor PA programs that provide safe and individualized outcomes.^16^ PT interventions for individuals with IDs can improve clients’ health in cardiovascular functioning, strength, and balance.^17^, ^18^

OTs incorporate knowledge of clients’ physical disabilities, including body functions and structures that decline with aging, to inform decision-making and implement recommendations for assistive devices that may impact PA.^19^ For example, in one study, OTs and OT students trained caregivers in the use of a virtual reality system that effectively promoted PA in adults with MMIDs and severe IDs.^20^ Other technologies, such as smartphone reminders to engage in PA used with children in schools, have shown promise in adult populations. ^21^ OTs might also promote increased independence in a patient’s care routine and mobility and improve health knowledge and empowerment, which may impact PA.^22^

Nurses, doctors, respiratory therapists, speech-language pathologists, and other clinicians directly or indirectly attend to cardio-respiratory function, which may impact PA. As part of vital sign monitoring, respiratory rate is critical in predicting adverse events like cardiac arrest or overall physiological deterioration. 23, 24 Inhalers and nebulizers may assist with breathing function, and medications to treat gastroesophageal reflux may assist with coughing. Furthermore, clinicians may assist with proper positioning and swallowing evaluations to help prevent aspiration pneumonia.^25^ Nurse practitioners can encourage PA within routine care, worksite wellness, and school-based environments.^26^ As pain can deter PA, many clinical professionals monitor pain via pain scales and prescribe non-pharmacological or pharmacological interventions as appropriate within their scope of practice.^27^

Little is known regarding PA interventions focused on adults with SPIDs/MIs. Many individuals with SPIDs, especially adults with profound IDs who have concurrent MIs, must depend on others to support their participation in PA.^28^ For example, research shows the positive effects of bouncing ball exercises to increase alertness and the use of innovative therapies like robotic exercise machines to enhance movement.^29^ Incorporating aquatic therapies has also shown to be a well-accepted form of PA for people with SPIDs/MIs.^30^, ^31^, ^32^

## 2 Methods

### 2.1 Purpose/aim/research questions

This study aimed to investigate the lived experience of PA in adults with SPID/MI from the perspective of clinicians who support these individuals. The primary research question was: How do individuals with SPIDs/MIs experience PA as seen from the perspective of clinicians?

### 2.2 Study design and participant recruitment

This study utilized a phenomenological qualitative research design to explore the experiences and perceptions regarding PA in adults with SPIDs/MIs from the perspectives of clinicians. The study was conducted between March and November 2023 and used convenience, purposeful, and snowball sampling methods. All procedures were performed in compliance with relevant laws and guidelines, with Institutional Review Board approval for this study provided on March 8th, 2023. Research data has not been shared within a data repository to protect the confidentiality of participants. The privacy rights of human subjects have been observed and consent was obtained via informed consent.

Participants were recruited via word of mouth, personal social media accounts, personal and professional connections, the American Association of Intellectual and Developmental Disabilities’ and the National Center on Health, Physical Activity, and Disability’s social media, and the National Association of County and City Health Officials’ monthly disability newsletter.

Potential participants completed a Qualtrics® survey to determine eligibility for the study, followed by a scripted phone screen by the primary investigator (PI). To be eligible for the study, participants must have been clinicians for individual(s) with a severe or profound intellectual disability. Examples of clinicians who would likely qualify for participation included occupational therapists, physical therapists, nurses, registered dietitians, and adaptive exercise experts. Adapted exercise experts included certified adapted physical educators, recreation program developers, therapeutic recreation specialists, certified inclusive fitness trainers, or those with other adapted exercise certifications. Another inclusion criterion was that clinician participants must have worked with the individual(s) with the disability who: (a) was an adult (at least 21 years of age or older [and no longer receiving school-based education under an IEP]), (b) had a medically documented diagnosis of severe or profound intellectual disability diagnosed during the developmental period (before age 22) per family caregiver report, (c) required the use of a wheelchair as the primary mode of mobility as per family caregiver report, and (d) resided at home with a family caregiver(s). Exclusion criteria included having less than 1 year of experience working as a clinician and not residing in the U.S.

### 2.3 Data collection and analysis

Twenty-nine individuals completed the initial Qualtrics® survey screening, 9 met the criteria for phone screening, and 8 of these 9 individuals agreed to participate in the study by signing an informed consent form. To ensure confidentiality, participants were provided with a pseudonym via a coding system. For example, the first clinician participant was designated as Clinician 1, or C1. Demographic information was collected as part of the interview process. Qualitative data were collected through semi-structured interviews via Zoom, utilizing an interview guide with questions based on research studies exploring PA among those with SPIDs. The interview guide was reviewed by a qualitative research expert to ensure the questions were bias-free. Interview questions were organized according to the five levels within the socio-ecological model (SEM) to promote confirmability and trustworthiness (Table 1). As there is no universal description of how PA manifests in adults with SPIDs/MIs, participants were encouraged to describe their perception and experience as it related to PA engagement in their clients with SPID/MI. Interviews lasted approximately one hour. After each interview, the verbatim transcription of the Zoom audio recording was uploaded and manually coded within ATLAS.ti (Mac version 23.2.1)^33^ qualitative software for analysis.

**Table 1:** Clinician interview guide.

**Clinician Interview Questions**	**Evidence or rationale to support question (s)**
*Warm up* • Thank you for taking time to speak with me today. As described in the consent form, I am interested in learning about your experience as a clinician working with ____________ in terms of promoting engagement in physical activity. • When you think of the term “physical activity” in reference to people with disabilities or those who use a wheelchair what comes to mind? • When you think of physical activity for _____________is this definition different? How so?	To build rapport and understand the “lived experience” of the participant
*(Intrapersonal domain)* • I’d like to understand your perspective about working with clients who have severe or profound intellectual disability (SPID) and use a wheelchair. • What factors within the person (and specifically ___________) seem to influence engagement in physical activity? • Can you talk about the environmental context within your client (_________) or most of your clients that seem to influence engagement in physical activity. For example, discuss contextual factors like physical, cognitive, social factors related to _______ that hinders/helps engagement in physical activity. • When you are working with ___________ or other clients, what types of recommendations/interventions do you make to promote engagement in physical activity? • When you think about working with __________ and trying to promote physical activity (given their current limitations) please discuss your overall assessment/ satisfaction with _________’s level of physical activity. *Probes*	To understand the “lived experience” of the participant 29, 31, 35, 37, 43
• Discuss barriers for involvement in physical activity that are internal to ___________. We’ll talk about factors that are external to _____ a little later.	
*(Interpersonal domain)* • In what ways (if any) have you worked with the caregiver and/or other professionals to promote engagement in physical activity? • In what ways (if any) do you see interpersonal relationships within a family/friend group being used to promote physical activity? • Please discuss the ways (if any) that other people or relationships influence engagement in physical activity. o When you think about other people (including yourself) are there ways that they support engagement in physical activity? o Are there people or interactions that detract from or hinder engagement in physical activity? • In relation to _________ what people or groups of people have been or might be used to promote engagement in physical activity?	To understand the “lived experience” of interacting with others in relation to physical activity^29^, ^31^, ^35^, ^37^, ^43^, ^54^
*(Organizational domain)* • Please discuss any involvement (yours and __________’s) in community organizations that might provide opportunities for movement or physical activity. • In what ways (if any) do these organizations influence engagement in physical activity? • Do these organizations host regular events? • Do these organizations provide regular communication or educational opportunities? • If you were talking to a family member of a person with a disability, what organizations (you don’t have to give specific names…. just the type of organization) would you say tend to support engagement in physical activity? Are there organizations that you would not recommend?	To understand the potential influence of how organizations influence engagement in physical activity^29^, ^35^, ^37^
*(Community domain)* • Please discuss the community environment for ____________ or your other clients. • Rural, Suburban, Urban? • Are their places to go safely outside? • What is the transportation/traffic situation in your area? • Tell me about your community and recreational opportunities. o Gyms, parks, walking trails? o Are parks and/or classes open and accessible to people in wheelchairs? • In what ways (if any) does the community influence (both positive and negative) __________ ‘s involvement in physical activity or movement?	To understand the potential influence of the community influences engagement in physical activity^17^, ^29^, ^35^, ^37^, ^52^, ^53^
*(Public policy domain)* • Please tell me about any advocacy efforts (including advocacy via social media), laws or policies, that has (or has potential to) influence your client’s engagement in physical activity. *Probes* • Comment on the level of funding for programs for people in wheelchairs or those with more involved intellectual impairments. • Comment on any recent advocacy efforts (for or against) people with disabilities being more involved in physical activity (For example, a group may have advocated for swings that have wheelchair ramps built into them to enable people with disabilities to get physical activity and be outside). • Let’s pretend that you have the opportunity to testify before a legislative panel (at the county, state, or federal level). What funding or policies would you advocate for to promote physical activity for persons with disability?	To understand the potential influences of how public policies might influence engagement in physical activity^35^, ^52^
*Dream phase:* • What do you wish others (including healthcare providers) knew about people with SPIDs who use a wheelchair and their potential engagement in physical activity? • If you were in “charge of the world” what changes might you implement to enhance opportunities for people with SPID who use a wheelchair to be more physically active?	To promote collaboration with the participant caregivers in terms of influencing practice and policy for persons with disability

To ensure trustworthiness, member checking was conducted by providing clinician participants with a summary of themes from their transcript to review for accuracy. Field notes were used to corroborate and supplement themes. Additionally, as a form of bracketing, the PI maintained a reflexivity journal to log values and opinions regarding participants’ statements so that they remained separated from the meaning analyzed from the data. This reflexivity journal also outlined the decision-making process and procedures for identifying and categorizing themes. Finally, a peer debriefer with expertise in qualitative research reviewed the transcripts and results obtained by the PI and confirmed the dependability of the findings.

## 3 Results

### 3.1 Participant demographics

Data saturation was achieved via 8 clinician participants. Seven participants self-identified as female, and 1 participant self-identified as male. Participants were between years old, with a range of 4–25 years of clinical experience. Seven participants were White, and 1 participant was Afro-Caribbean. Participant professions included speech-language pathology (n = 1), occupational therapy (n = 2), physical therapy (n = 2), certified fitness instructor (n = 1), adaptive fitness manager (n = 1), and physician (n = 1). Participants were asked to think of a specific client with whom they had worked to facilitate an understanding of specific client experiences. The clients (described by clinician participants) ranged between 21 and 68 years old. Five clients were male, and 3 were female. Five clients were White, 1 was Hispanic, and 2 were African American. Considering ID level diagnosis of the clients, 6 had severe ID, and 2 had profound ID. For a complete listing of all demographic data, please refer to Table 2.

**Table 2:** Demographic data.

**Clinician Participants (N = 8)**		
	**Clinicians**	**Clients**
**Age**	35–64	21–68
**Gender**	7 Female, 1 Male	3 Female, 5 Male
**State of Primary Residence**	New York, Georgia, Arizona, Texas, Florida, Missouri	New York, Georgia, Arizona, Texas, Florida, Missouri
**Years of Practice**	4–25 Years	
**Race/Ethnicity**	7 White/Non-Hispanic 1 African Caribbean	5 White/Non-Hispanic 2 Black 1 Hispanic
**Profession**	1 Speech Language Pathologist, 2 Occupational Therapists, 2 Physical Therapists, 1 Physician, 1 Certified Fitness Instructor, 1 Certified Group Fitness Trainer/Adapted Fitness Manager	
**Intellectual Disability**		6 Severe, 2 Profound
**Other Primary Diagnoses (if known)**		Scoliosis, Seizure Disorder (2), Cerebral Palsy (2)

^*Note:* The term “Clients” refers to one individual that the clinician was asked to use an example of a typical client presentation when answering each of the interview questions.^

### 3.2 Theoretical framework and overall themes

The SEM may benefit clinicians in several ways. For example, it may assist fitness experts by illustrating the importance of environmental factors when implementing PA recommendations.^34^ For this study, data were organized according to the 5 SEM levels as well as 16 a priori themes based closely on the work of McLeroy et al.^35^: (1) *intrapersonal:* attitudes, physical factors, knowledge, and values, (2) *interpersonal:* supports outside the home, supports within the home, and home environment considerations, (3) *organizational:* disability-inclusive organizations, academic institutions, and medical institutions, (4) *community:* environment and priorities, and (5) *policy:* home and community-based services (HCBS), financial, academic and programming, and accessibility policies. Clinician-identified barriers and facilitators to PA were grouped under these 16 themes.

Our qualitative analysis involved a dynamic and iterative combination of inductive and deductive coding. The process began with inductive coding, where we identified themes that emerged directly from the data, allowing us to uncover insights and patterns without preconceived notions. This initial exploration was followed by deductive coding, which was guided by the structural components of the SEM, such as knowledge, attitudes, supports, academic institutions, and policies. However, the process was not strictly linear; it involved multiple rounds of sorting and sifting through the data. We continuously revisited the codes, alternating between inductive discovery and deductive application. This iterative back-and-forth allowed the SEM framework to integrate and reflect the emergent findings while maintaining its theoretical grounding.

Given the extensive number of themes and subthemes identified within the study’s results, only the top five barrier and facilitator subthemes are discussed (Table 3). For a complete listing of all themes, subtheme barriers and facilitators, and the number of associated responses for each theme and subtheme, please refer to Supplementary Materials.

**Table 3:** Most frequent barriers and facilitators to physical activity (PA) utilizing the socio-ecological model (SEM).

**SEM Level**	**Themes**	**Barriers**	**Supporting Quotes**	
Community	Environment	#1 Building, Outdoor, & Transportation Inaccessibility	**C6:** “We live in the oldest city in the country, and because it's historical, it does not necessarily have to be accessible. So, for her [client with SPID/MI] to access the downtown, I ventured to guess she doesn't really go downtown, which is, where a lot of activity happens.”	
Intrapersonal	Knowledge	#2 Necessity of Education on Needs and Opportunities for Physical Activity	**C4:** “I think people need to look for opportunities in the daily routine that are already there. If you're getting ready, right after bathing, you tend to be more relaxed. Maybe muscles are looser. That'd be a great time to add that. So, with any kind of new thing, it seems to be most successful if you attach it to what you already do successfully. It kind of gives you a boost of confidence, and it helps to prompt that as part of the routine.”	
Intrapersonal	Physical Factors	#3 Care Recipients’ Diagnoses, Bodily Structure, Pain, or Weakness	**C6:** “Her [muscle] tone and physicality…she's dependent for all transfers…that influences her ability to be moving and using those muscle groups.”	
Policy	Financial Policies	#4 Lack of Billing Structures to Support Physical Activity Programs and Clinician Reimbursement	**C4:** “A lot of those services, both from the school, and both from the private therapies are not continued after they [people with SPIDs/MIs] reach adulthood, because they're no longer services that are paid for by public insurance, unless they have a home and community-based state waiver that can cover some of those ancillary services for PT, OT, and speech [therapy].”	
Policy	Home & Community Based Service (HCBS) Policies	#5 Lack of HCBS Education and Support	**C2:** “I've been a part of this [HCBS waiver waitlists] with parents and they've said their kids might not get something unless somebody else's kid dies.”	
		**Facilitators**		
Intrapersonal	Attitudes	#1 Physical Activity Preferences Uniquely Individualized	**C7:** “It [PA] has to be individualized. It cannot be generalized…it has to do with their home environment and also the level of their disability.”	
Organizational	Disability-Inclusive Organizations	#2 Providing Activities for Adults with Multiple Disabilities	**C3:** “A lot of our churches around here have paired up with different nonprofit organizations…it's not every single week, but they do something probably at least once a month, or, if not, a couple of times a month, so definitely look into church organizations and local nonprofits.”	
Interpersonal	Supports Outside Home	#3 Importance of Allyship & Socialization (Adults with Disabilities & Caregivers)	**C8:** “A lot of times it might be family members, whether that's parents or siblings [who encourage PA] …it's often encouraging siblings to help engage [adults with SPIDs/MIs] in physical activity and there's a social element to that that I think can be really helpful sometimes.”	
Community	Environment	#4 Building, Outdoor, and Transportation Accessibility	**C3:** “We raised funds for a van…We will use it on Saturday for a race. We have a volunteer that's going to go pick up 2 of our wheelchair participants and get them to the race.”	
Policy	Financial Policies	#5 Advocacy for Promoting Monies Toward Prevention Instead of Illness	**C5:** “If somebody's in a defined movement program of any type, why wouldn’t Medicaid reimburse for that at some level? If we give people the opportunity to eat right and to move well, other [medical] costs will go down.”	

### 3.3 Barriers and facilitators to PA organized by SEM levels

#### 3.3.1 Intrapersonal

Four principal themes emerged within the intrapersonal level of the SEM: (1) attitudes, (2) physical factors, (3) knowledge, and (4) values. When considering all themes reported within this study, attitudes were the most frequently cited theme among clinician participants. Within the theme of attitudes, PA preferences being uniquely individualized was also the highest reported PA facilitator among the study’s overall results (Supplementary Materials). One occupational therapist participant (C6) described her clients’ interest (attitude) in adapted surfing. She stated:

*“They [local organization] do a day once a month, where they meet at the beach, and they have a ton of volunteers as well as some expert local surfers and they use a huge board. Five people get in the water and help push the surfboard, and they have surfboards where people can sit in like a chair. It's almost like a pontoon boat, and they can surf on that. It's really neat, actually.”*

Overall, participants identified numerous types of PA. However, the most frequently reported PA types were passive range of motion/stretching, standing with assistance, fitness classes, and aquatic activities.

The necessity of education on the needs and opportunities for PA as well as client factors, such a diagnoses, bodily structure, pain, or weakness were the second and third most cited barriers to PA identified by clinicians. A certified fitness instructor participant (C5) described the need for training fitness professionals/business owners and its potential impact on PA opportunities:

*“This population [of adults with IDs/MIs] is massive. People don't realize that… And even from an economic standpoint, what I tell other fitness organizations is that there's so much opportunity here. There are caregivers that would pay for you to work with them… It's an opportunity to serve the people and…it’s awareness… If we do action with awareness then the needle starts to move, right? [to help improve PA for adults with disabilities].”*

An adapted fitness manager (C3) described her work with an individual with SPID/MI and the caregiver’s struggle in maintaining PA given the client’s diagnoses:

*“She [caregiver] is always making sure he [son] is engaged and moving and doing things and trying to keep him doing things for himself as much as possible. He's in a wheelchair, but she'll [caregiver] get him up and walk him. She holds him by the belt, and he can move his legs. So, she's out there trying to get him moving…He just has a very, very difficult time with balance and with the seizures. If he has one [seizure], he'll fall. And yeah, he's knocked a tooth out and everything and [he] bruised up [his] little eye. And it's just terrible.”*

#### 3.3.2 Interpersonal

Three principal themes emerged within the interpersonal level of the SEM: (1) supports outside of the home, (2) supports within the home, and (3) home environment considerations. The importance of allyship and socialization for adults with SPIDs/MIs as well as for their caregivers (both under the theme of support outside of the home) was the third most frequent PA facilitator noted by participants. Regarding the impact of socialization on PA, C5 stated:

*“One moment, I got a video where she [fitness class participant] got in her chair, and she tried to stand up because she wanted to be like the other people… I think with the right encouragement, in the right environment, they [adults with SPIDs/MIs] may do things that we haven't seen or knew that they would even want to try…I can't describe to you exactly why this happens… In that person's mind, I have to think personally that they see everybody and their peers exercising. They see it. They're having fun. They feel some pride and it's like they're going to show us, and we see similar things like that repeatedly.”*

#### 3.3.3 Organizational

Organizational-level themes found included (1) disability-inclusive organizations, (2) academic institutions (i.e., colleges providing internships for clinicians to work with adults with SPIDs/MIs), and (3) medical institutions. Organizations that provide PA and PA funding for adults with multiple disabilities (under the theme of disability-inclusive organizations) were the study's second most prevalent PA facilitators. C6 described her positive experience with local organizations aiming to provide inclusive PA programs for those with SPIDs/MIs:

*“I might also recommend to a parent or caregiver that they look at organizations in the area, like the Cerebral Palsy League, or Easter Seals, or March of Dimes, and a lot of the stuff seems to be very grassroots, and you have to look to your grassroots organizations to see what they're doing, because the stuff out there, it's just hard to find.”*

#### 3.3.4 Community

There were two primary SEM community-level themes: (1) environment and (2) priorities. Building, outdoor, and transportation accessibility and building, outdoor, and transportation inaccessibility (under the theme of environment) were the 4th highest facilitator and highest reported barrier within this study, respectively. Regarding the facilitator of building, outdoor, and transportation accessibility, a physician participant (C4) specifically described the importance of universal design:

“It should just be commonplace for kids and adults to play on accessible playgrounds. If you do it by universal design, it doesn't have to be the exclusive thing. It can be inclusive.”

Regarding building, outdoor, and transportation inaccessibility, a speech-language pathologist (C1) discussed multiple barriers:

*“Not everything is handicapped accessible. There are difficulties, like say the person uses the bathroom. How would you take them into the restroom, say, at the baseball game? Is the handicapped stall big enough or could they get up? Maybe there's no ramps or the sidewalks aren’t approved for wheelchairs. Maybe the doors weren't wide enough, or their van broke. Yeah, that's another issue. I see a lot. Their handicapped accessible vans break down. The wheelchair thing to put the wheelchair in the van doesn't work. It's not such an easy fix to take it to a mechanic. These people [with disabilities] have difficulty dealing with the wheelchair manufacturers, and there's just not enough of those people [wheelchair manufacturers], especially in this area. I'm sure it's everywhere. But it could take 6 months to get someone to come from a wheelchair company to help, say, like a broken pedal or a break lock, so maybe you can't put the brakes on which would be dangerous when they're out.”*

#### 3.3.5 Policy

Finally, four SEM policy-level themes originated from the study: (1) home and community-based service (HCBS) policies, (2) financial policies, (3) academic and program policies (i.e., schools and day programs), and (4) accessibility policies. Advocacy for promoting monies toward prevention and illness (under the theme of financial policies) was the 5th highest facilitator of PA identified. A physical therapist (C8) provided an example of the importance of prevention:

*“Probably the first thing I would be advocating for is improved funding for therapy services because in my professional opinion, I think individuals with lifelong and profound disability really need more services that are skilled services throughout their life to help maintain their physical wellness, which can make a huge difference on overall global health and it's less expensive upfront than, you know, the medical expenses can be if people aren't addressing some of those issues.”*

C3 also spoke on this topic:

*“We have Medicaid and SSI and stuff for our people with disabilities, but [not] something that would pay for their physical fitness, and for classes for them to attend. Could they take some of that? That's only used for going to the doctor for illness. And instead of…maybe if we're able to get them moving and talking to them about healthy eating and that type of thing, we wouldn't have to use so much of their money for going to the doctor, because what ends up happening…so someone that's in a wheelchair, they're not as active. They're not engaged in the community. Oh, well, now, all of a sudden, we're overweight. Oh, well, because we're overweight, we have heart problems. Oh, we have blood pressure problems. Oh, we have blood sugar problems, where, if we're active and moving and eating properly, those things aren't going to happen on top of what they're already dealing with.”*

Lack of a competitive billing structure to support PA programs and clinician reimbursement (under the theme of financial policies) and lack of HCBS education and support (under the theme of HCBS policies) were the 4th and 5th most noted barriers to PA. Another physical therapist participant (C7) spoke of the limitations of her 30-minute sessions with clients regarding billing and reimbursement:

*“You only have a 30-minute session with them [adults with SPIDs/MIs], and within that 30-minute session, you still have to take time to document that communication, so your heart is working, and you want to give your best, but again, you are taking time from you as well, because policies and everything expect you to really do all the documentation that is required.”*

Finally, to demonstrate the lack of HCBS education and support for services needed, an occupational therapist participant (C2) mentioned:

*“I think what I would ask for is for part of the monies to be used on education of what is available because I think so many of these parents are detectives. And the OTs [occupational therapists] and PTs [physical therapists] …some of us are tapped…but it is detective work. So, I would actually ask for part of the [HCBS] money to be used on education, Clearinghouse, or websites, so that people know what is available.”*

For additional supporting quotes related to the discussed barriers and facilitators to PA among adults with SPIDs/MIs, please refer to Table 3.

## 4 Discussion

### 4.1 Intrapersonal

This qualitative study was the first to focus on barriers and facilitators to PA among adults with SPIDs/MIs from the perspective of clinicians. The abundance of themes and subthemes among all five SEM levels demonstrated the interplay of how barriers and facilitators may increase or decrease PA opportunities. The most frequently reported PA facilitator was PA preferences as uniquely individualized. This facilitator is unsurprising as patient preferences are typically linked to adherence and satisfaction with PA in the general population.^36^ It is also likely that engaging in preferred activities encourages an element of fun (an additional facilitator within the community SEM level of this study), as observed in other studies examining PA among those with IDDs.^37^, ^38^

Moreover, this study reported range of motion/stretching as the most popular PA type identified by participants working with adults with SPIDs/MIs. While range of motion and flexibility are important, these exercises may act as a precursor to PA and may not necessarily equate to the 150–300 minutes of moderate-intensity PA recommended by the U.S. DHHS^7^. In contrast, other studies indicated that walking is the primary source of PA for those with IDs.^39^, ^40^ Nakken and Vlaskamp^41^ reported that people with profound IDs frequently had limitations in mobility, requiring them to use wheelchairs or other assistive devices, thus demonstrating evidence to support diagnoses, bodily structure, pain, or weakness as a barrier to PA within this study. Dixon-Ibarra^38^ further corroborated the negative impact of comorbidities on PA participation.

The necessity of education on needs and opportunities for PA was also a significant hindrance to PA in this study. Many participants reported that other fitness professionals or direct care staff may need help understanding the benefits or PA needs among those with SPIDs/MIs, a finding seen in multiple studies.^38^, ^40^ It should also be noted that this study did not identify the quantity of physical activity as seen with more structured activities. As such, non-structured, stereotypical movement disorders are sometimes observed among individuals with IDs and include repetitive motor behaviors such as body rocking, hand flapping, and hitting.^42^ Some staff may not routinely identify these activities as PA, further demonstrating the impact of knowledge deficits on opportunities for PA.

### 4.2 Interpersonal and organizational

Considering the interpersonal domain of the SEM and supports outside of the home, the importance of allyship and socialization for adults with disabilities and their caregivers was a significant facilitator to PA. Participation in a group or team, such as group fitness classes, was highly suggestive of PA participation in our study, similar to other studies focused on adults with IDDs.^37^, ^38^, ^40^ Our findings also suggest that socialization may have instilled a sense of camaraderie. Taliaferro and Hammond ^43^ reported that primary caregivers, siblings, friends, and others supporting PA were primary facilitators in PA participation. Additionally, another finding within this study was the potential promotion of PA by connecting caregivers with other caregivers to help facilitate discussions about PA opportunities and shared experiences. Disability-inclusive organizations that provide PA conducive to the needs of adults with multiple disabilities, like SPIDs/MIs, are crucial facilitators in connecting both adults with disabilities and caregivers to others with these shared, lived experiences. Schools may consider discussing physical activity opportunities during age-related transition planning during IEP meetings. The discontinuation of programs focused on PA among those in this group was a barrier identified in previous studies.^44^ However, this was not a barrier identified by clinician participants in this research, suggesting that PA programs for those with SPIDs/MIs do exist and that caregivers would benefit from direction as to how to identify and engage their care recipient in these programs during school to adult service transition processes.

### 4.3 Community

Building, outdoor, and transportation accessibility and inaccessibility were facilitators and barriers within this study, respectively. Transportation difficulties due to location from PA programs, public access, and the overall geographical landscape identified within this study were synonymous with other study results.^43^ Conversely, promoting accessibility through universal design for all healthcare programs and services was an example of a method to improve PA within this study and other literature and might include inappropriate visual signage or demonstrations for those with disabilities.^26^

### 4.4 Policy

Regarding financial policies, advocacy for promoting monies toward prevention instead of illness was a significant facilitator to PA, while the lack of a competitive billing structure to support PA programs and clinician reimbursement was a primary barrier to PA. Evidence suggests that financial incentives can appropriately target health outcomes and health equity.^45^ Despite this, no research explicitly exists regarding monies focused on prevention instead of illness for those with SPIDs/MIs. Furthermore, tensions still exist among many providers and those with disabilities to differentiate between the medical model and the social model of disability. The medical model suggests that disability is something to be cured, and the social model is a form of valued diversity.^46^

Billing structures aimed at streamlining provider visits to promote competitive compensation for services may incentivize more providers to provide PA services for adults with SPIDs/MIs. In one study, adults with cerebral palsy seeking health services found it difficult when transitioning from pediatric to adult rehabilitation as there was little or no funding for services such as PT or OT for this group, and fee-for-service payment systems did not provide adequate compensation for clinicians.^47^

Finally, this study found that lack of HCBS education and support was a significant barrier to PA participation. Regarding PA provision, Medicaid provides mandatory services such as physician coverage, inpatient and outpatient hospital services, transportation to medical care, and x-rays. Optional services include speech therapy, OT, PT, rehabilitative services, respiratory care, eyeglasses, personal care, HCBS waiver services, and other community-based supports.^48^ If these services are “optional,” fewer opportunities for those with ID to engage in physical activities may exist. For example, in community-based settings, a recent study evaluated Medicaid claims related to PT and OT usage for adults with and without cerebral palsy with a musculoskeletal diagnosis. Results indicated that, in older adults with a diagnosed musculoskeletal disorder, less than one-third used PT and OT services.^49^

Only twenty-five state service plans mentioned utilizing the HCBS waiver to provide long-term PT care for chronic conditions instead of short-term acute care, and 26 state service plans allowed HCBS waiver PT services provision in patients’ homes or communities.^50^ Additionally, the provision of PT interventions under HCBS waivers varies significantly by state. Based on 2015 data, utilization of PT services ranged from 1 to 1215 participants. Of importance is that, in some states, HCBS waiver services for PT include caregiver or staff training to implement the recommendations made by physical therapists in the home environment.^50^, ^51^ These healthcare access concerns may be an essential consideration for adults with SPIDs/MIs transitioning and aging in the family care environment.

## 5 Recommendations for future research

The results of this study suggest further opportunities for research. For example, regarding physical factors related to PA with the intrapersonal level, sleep/alertness patterns determining the timing of PA was a barrier to PA reported among participants. Future research studies should involve PA interventions as part of a structured program incorporating individualized alertness patterns to determine their impact on PA quantity and other health outcomes.

Within the interpersonal SEM level, as previously discussed, participants reported a high response to the importance of allyship and socialization among people with disabilities and caregivers. Future studies should include exercise interventions to promote group activities incorporating multiple caregivers and adults with SPIDs/MIs.

Two of the most highly reported themes, building, outdoor, and transportation accessibility and inaccessibility, present an enormous opportunity for future research. For example, studying the impact of accessible ride-shares or transportation services among members of PA programs may provide valuable insight into the feasibility of these services and a better understanding of how they impact health outcomes and future health costs. Moreover, from a policy perspective, exploring the financial and health benefits of preventative practices like PA programs and therapies should be evaluated in longitudinal studies to determine if savings and improved health outcomes are achievable via this method versus a focus on treating illness.

## 6 Strengths and limitations

This study had numerous strengths. First, trustworthiness took many forms. For example, dependability was maintained via the primary investigator’s bracketing of experiences/opinions before interviews through reflexive journaling and semi-structured interview guides. Credibility was ensured via theoretical triangulation using the SEM, data source triangulation between interview responses and observational data, and method triangulation through interviews, field notes, and observations. Confirmability was maintained via multiple quotes to support themes, and to promote transferability, participants resided in various states throughout the U.S. and represented vast age groups and clinical experience. Clinicians worked across multiple health professions, such as PT, OT, adapted fitness, speech-language pathology, and medicine. Clients also represented different age groups, ethnic backgrounds, locations, and severe or profound IDs.

There were three potential limitations of this study. Despite three attempts, 1 of 8 participants did not respond to multiple attempts at member checking following their interview. Also, it should be noted that clinicians were asked to focus on one client who met the study criteria. Some clinicians frequently referred to more than one client. Although this enhances the transferability of the study, it is uncertain whether focusing on multiple clients was a strength or a limitation. Finally, although the study was diverse in many respects, it included only one male clinician participant. Additional male perspectives might have provided alternative insights and further enriched the study’s results.

## 7 Conclusions

In summary, clinicians perceive various barriers and facilitators to PA among adults with SPIDs/MIs. Using the five components of the SEM (intrapersonal, interpersonal, organizational, community, and policy), understanding barriers and facilitators may help families, clinicians, policymakers, and community collaborators increase PA in adults with SPIDs/MIs, particularly those individuals who are transitioning to adult and community-based settings.

## CRediT authorship contribution statement

**Chandler Judy:** Writing – review & editing. **Chatto Charlotte:** Writing – review & editing. **Shotwell Mary:** Writing – review & editing, Supervision, Methodology, Formal analysis, Conceptualization. **Perkoski Valerie:** Writing – review & editing, Writing – original draft, Methodology, Formal analysis, Data curation, Conceptualization.

## Ethical statement

We have included a statement within the manuscript that informed consent was obtained by participants to engage in human subjects’ research. The privacy rights of human subjects were and must always be observed.

## Funding

This research did not receive any specific grant from funding agencies in the public, commercial, or not-for-profit sectors.

## Declaration of Competing Interest

The authors declare that they have no known competing financial interests or personal relationships that could have appeared to influence the work reported in this paper.

## Data Availability

The data that has been used is confidential.
