# Supplementary material for: Pushing forward: Understanding physical activity in adults with medical complexity
Source: Health Care Transit. 2025 Apr 18;3:100102. doi: 10.1016/j.hctj.2025.100102 (PMC12033943; doi:10.1016/j.hctj.2025.100102)
Supplement: Supplementary file 1 — Supplementary material [file mmc1.docx]

**Supplemental.** Themes and Subthemes Utilizing the SEM Framework.

| **SEM**  **Level** | **Principal Theme** | **Sub-Theme**  **(Barriers)** | **Sub-Theme**  **(Facilitators)** |
| --- | --- | --- | --- |
|  | ***Attitudes*** *(271)* | Clinician/Caregiver Fear of Causing Injury (16) | PA Preferences Uniquely Individualized (165) |
|  |  | Unwillingness to Engage with People with Disabilities/Attitudes (10) | Caregiver Receptivity and Promotion of PA (36) |
|  |  | Clinician/Caregiver/Staff Burnout (9) | Presuming Competency/Capability of Participating in PA (19) |
|  |  | Lack of PA Opportunities (5) | Prioritizing Caregivers’ PA (5) |
|  |  |  | Positive Impact on Those who Assist with PA (3) |
|  |  | Expectation Placed on Caregivers to Teach/Continue PA after Therapy Services End (1) | Persons with Disabilities’ Desire to Please/Show Others (2) |
| **Intrapersonal** | ***Physical***  ***Factors*** *(134)* | [Clients’] Diagnoses, Bodily Structure, Pain, or Weakness (25) | Importance of Positioning or Transfer Devices (38) |
|  |  | Physical Difficulty & Lack of Assistance with Transferring Person w/ Disability/Aging Caregivers (9) | Importance of PA for Promotion of Health & Independence (19) |
|  |  | [Clients’] Dependence on Caregiver or Others (9) | Cognitive Capacity/Disability Severity (3) |
|  |  | Lack of PA (8) |  |
|  |  | Sleep/Alertness Patterns Determining Timing of PA (8) |  |
|  |  | Inability to Communicate (7) |  |
|  |  | Cognitive Capacity (3) |  |
|  |  | Lack of Focus (3) |  |
|  |  | Aversion to Sweating (2) |  |
|  | ***Knowledge*** *(66)* | Necessity of Education on Needs & Opportunities for PA (31) | Determining Motivators of PA for Person w/ Disability (20) |
|  |  |  | Creativity Importance (14) |
| **Intrapersonal** |  |  | Importance of Inquiring About PA Opportunities (1) |
|  | ***Values*** *(64)* | Caregiver Time & Work Restraints (5) | Acknowledging Choices, Needs, & Person-Directed Goals (27) |
|  |  |  | Connecting PA to Routine Activities/Family Engagement (21) |
|  |  |  | Feelings of Safety, Security, & Trust (6) |
|  |  |  | Sense of Belonging (5) |
|  | ***Supports Outside Home*** *(214)* | Social Challenges: (Inability to Communicate or Use Assistive Communication Devices (4) | Importance of Allyship & Socialization (People with Disabilities & Caregivers) (53) |
|  |  | Lack of Physician Involvement w/ Person’s PA (1) | Providing Music to Encourage PA (44) |
|  |  |  | Awards/Visual or Verbal Recognition (21) |
|  |  |  | Caregiver Promotion of PA (19) |
| **Interpersonal** |  |  | Attention to Verbal/Non-Verbal Cues & Enough Time to Respond (18) |
|  |  |  | Cheering, Clapping, or Celebrations (16) |
|  |  |  | Consistent Communication (12) |
|  |  |  | Encouraging PA Possibilities (9) |
|  |  |  | Ratio of Clinicians/Caregivers to Individuals w/ Disabilities (6) |
|  |  |  | Collaborating w/ Other Providers or Staff (6) |
|  |  |  | Providing Assistive Devices/Set-Up, or Modifications (4) |
| **Interpersonal** |  |  | Providers who Routinely Encourage PA (1) |
|  | ***Supports Within Home*** *(33)* | Fewer Opportunities for PA (1) | Virtual PA Classes/Platforms (13) |
|  |  |  | Caregiver Receptivity and Promotion of PA (12) |
|  |  |  | Encouraging PA Possibilities (3) |
|  |  |  | Verbal Recognition (2) |
|  |  |  | Therapies Provided in Home (1) |
|  |  |  | Attention to Verbal/Non-Verbal Cues & Enough Time to Respond (1) |
|  | ***Home Environment***  ***Considerations*** *(9)* | Lack of Assistive Devices or Accessible Layout/Set-Up (1) | Greater Comfort w/ PA Provided by Professionals in the Home (5) |
|  |  |  | Accessible Layout (3) |
|  | ***Disability-Inclusive Organizations*** *(66)* |  | Organizations that Provide PAs for Adults with Multiple Disabilities (66) |
| **Organizational** | ***Academic Institutions*** *(38)* | Need for Increases in Staffing to Support PA (10) | Student Internships/Education Working with Adults with IDDs (13) |
|  |  |  | Day Programs Providing Options/Supports for PA (10) |
|  |  |  | Ratio of Clinicians/Instructors to Individuals with Disabilities (5) |
|  | ***Medical Institutions*** *(15)* | Providers Not Providing Engagement or Support for PA (1) | Providers/Tools that Assess/Encourage Opportunities for PA (11) |
|  |  |  | Access to Innovative Therapies/Technology (3) |
|  | ***Environment*** *(204)* | Building, Outdoor, & Transportation Inaccessibility (61) | Building, Outdoor, & Transportation Accessibility  (63) |
|  |  | Noise (2) | Inclusivity Importance (38) |
|  |  | COVID-19 (2) | Colors or Sensory Considerations (15) |
|  |  | Transportation Cost (1) | Large Spaces (7) |
|  |  | Lack of Mechanics & Wheelchair Manufacturers (1) | Themed Workouts/Elements of Fun (7) |
| **Community** |  |  | Geographical Reach of Social Media in Promoting PA Programs (3) |
|  |  |  | Length of Activities (2) |
|  |  |  | Increased Virtual Communication Due to COVID-19 (1) |
|  |  |  | Advertising & Marketing Activities (1) |
|  | ***Priorities*** *(9)* | Cost to Improve Accessibility (1) | No or Low-Cost Opportunities (5) |
|  |  |  | Frequency of Activities and Opportunities Focused on PA (2) |
|  |  |  | Caregiver Advocacy (1) |
|  | ***HCBS***  ***Policies*** *(63)* | Lack of HCBS Education & Support (21) | Receiving HCBS Waiver Supports or Entitlements (7) |
|  |  | Waitlists for Services (19) | Waiver Funding Devoted to PA Programs and Therapies (2) |
|  |  | Transition to Adult Services (11) |  |
|  |  | Funding of Waivers (3) |  |
|  | ***Financial Policies*** *(101)* | Lack of a Competitive Billing Structure to Support PA Programs & Clinician Reimbursement (23) | Advocacy for Promoting Monies Toward Prevention Instead of Illness (45) |
|  |  | Transition to Adult Services (6) | Grant/Other Funding for Accessible Equipment (14) |
| **Policy** |  | Lack of Therapy Coverage Outside of Acute Injuries (6) | Financial Incentives at Fitness Centers (1) |
|  |  | Poor Pay for DSP and Others Working w/ those with IDDs (5) |  |
|  |  | Prioritizing Funding for Specific Diagnoses (1) |  |
|  | ***Academic & Program Policies*** *(7)* |  | Adaptive PA Exposure for Individuals & Family as Part of IEP or 504 Plan (4) |
|  |  | Staffing Concerns (3) |  |
|  | ***Accessibility Policies*** *(18)* | Lack of Universal Design/Meeting Bare Minimum (ADA) Requirements (7) | Statutes for Greater Accessibility/Universal Design (8) |
|  |  | Focusing on Accessibility, but Ignoring Usability (1) | Importance of Advocating at a Local Level (1) |
|  |  |  | Monies Allocated to Increase Accessibility (1) |
|  | **16 Themes** | **40 Sub-Themes (Barriers)** | **63 Sub-Themes (Facilitators)** |

*^Note:^* ^ADA, Americans with Disabilities Act; DSP, Direct Support Professional; HCBS, Home & Community-Based Services; IDDs, Intellectual & Developmental Disabilities; IEP,^ ^Individualized Education Plan; PA, Physical Activity; PT, Physical Therapy; OT, Occupational Therapy; SEM, Socio-Ecological Model. Numerical values in parenthesis reflect number of times themes and subthemes were reported by participants.^
